# Arsenic Levels in Chicken

**DOI:** 10.1289/ehp.1307083

**Published:** 2013-09-01

**Authors:** Tamar Lasky

**Affiliations:** MIE Resources, Kingston, Rhode Island, USA, E-mail: tlasky@mie-epi.com

I commend Nachman et al. (2013) on their careful study of arsenic content in market samples of chicken and am dismayed to learn that arsenic levels in chicken remain high despite our report of arsenic levels in *Environmental Health Perspectives* in 2004 (Lasky et al. 2004).

One possible explanation is the very complicated lines of authority around the regulation of drugs fed to food animals, along with the enforcement of those regulations. Nachman et al. (2013) focused their discussion on the role of the Food and Drug Administration (FDA), but many other agencies participate in the regulation and enforcement of residue safety in food animals. As noted by the Food Safety and Inspection Service (FSIS) in the introduction to their 2012 Residue Sampling Plans (FSIS 2012),

The U.S. National Residue Program (NRP) for Meat, Poultry, and Egg Products, administered by the USDA [U.S. Department of Agriculture] FSIS, is an interagency program designed to identify, rank and test for chemical contaminants in meat, poultry, and egg products.

They continue,

The NRP requires the cooperation and collaboration of several agencies for its successful design and implementation. The USDA FSIS, the EPA [U.S. Environmental Protection Agency], and the Department of Health and Human Services (DHHS) FDA are the primary federal agencies managing this program. The FDA, under the Federal Food, Drug, and Cosmetic Act, establishes tolerances for veterinary drugs, and action levels for food additives and environmental contaminants. The EPA, under the Federal Insecticide, Fungicide, and Rodenticide Act (as modified by the Food Quality Protection Act), establishes tolerance levels for registered pesticides…. Representatives from FSIS, FDA, EPA, the USDA Agricultural Research Service (ARS), the USDA Agricultural Marketing Service (AMS), and the DHHS Centers for Disease Control and Prevention (CDC) collaborate to develop the scheduled sampling program.

Setting and enforcing safety levels involves several steps, one of which is the collection of meat and poultry samples, followed by statistical analysis, interpretation, and action. The NRP sampling plan is designed to identify samples with residues above the allowed levels. The data are then analyzed as categorical values (violation, no violation). Current methods of data analysis do not include estimation of mean values that can then be extrapolated to the national food supply. It was by analyzing the data as a continuous variable that my coauthors and I were able to describe the high levels of arsenic in chicken in 2004 (Lasky et al. 2004).

Concerns about arsenic levels in chicken are of increasing importance because of the increased per capita consumption of chicken over the past decade (USDA Economic Research Service 2013).
